# Acute pharyngitis in children and adults: descriptive comparison of current recommendations from national and international guidelines and future perspectives

**DOI:** 10.1007/s00431-023-05211-w

**Published:** 2023-10-11

**Authors:** Roberta Pellegrino, Edoardo Timitilli, Maria Carmen Verga, Alfredo Guarino, Iride Dello Iacono, Immacolata Scotese, Giovanna Tezza, Giulio Dinardo, Simona Riccio, Sofia Pellizzari, Sonia Iavarone, Giulia Lorenzetti, Giovanni Simeone, Marcello Bergamini, Daniele Donà, Luca Pierantoni, Silvia Garazzino, Susanna Esposito, Elisabetta Venturini, Guido Castelli Gattinara, Andrea Lo Vecchio, Gian Luigi Marseglia, Giuseppe Di Mauro, Nicola Principi, Luisa Galli, Elena Chiappini, Other members of the Italian Panel for the Management of Acute Pharyngitis in Children, Stefania Stefani, Giulia Brigadoi, Fabio Midulla, Claudio Cricelli, Luigi Terracciano, Annalisa Capuano, Eugenia Bruzzese, Daniele Ghiglioni, Fusani Lara, Eleonora Fusco, Paolo Biasci, Lamberto Reggiani, Mattia Doria, Eugenia Bruzzese, Luigi Matera, Enrica Mancino, Elisa Barbieri, Antonio D’Avino, Laura Cursi, Maria Giuseppa Sullo, Silvestro Scotti

**Affiliations:** 1 Department of Health Sciences, Postgraduate School of Pediatrics, University of Florence, Florence, Italy; 2 ASL Salerno, 84019 Vietri Sul Mare, Salerno, Italy; 3 Department of Translational Medical Sciences, Federico II University, Naples, Italy; 4 Unit of Allergology, Division of Internal Medicine, Fatebenefratelli Hospital, Benevento, Italy; 5 ASL Salerno, 84022 Campagna, Italy; 6 Department of Pediatrics, Ospedale San Maurizio, Bolzano, Italy; 7 Department of Woman, Child and of General and Specialized Surgery, University of Campania ‘Luigi Vanvitelli’, Naples, Italy; 8 Department of Surgical Sciences, Dentistry, Gynecology and Pediatrics, Pediatric Clinic, University of Verona, Verona, Italy; 9 Department of Maternal, Infantile, and Urological Sciences, “Sapienza” University of Rome, Rome, Italy; 10 Residency School of Pediatrics, University of Rome Tor Vergata, Rome, Italy; 11 ASL Brindisi, 72023 Mesagne, Brindisi, Italy; 12 AUSL Ferrara, 44121 Ferrara, Italy; 13 Division of Paediatric Infectious Diseases, Department of Women’s and Children’s Health, University of Padua, Padua, Italy; 14 Pediatric Emergency Unit, IRCCS Azienda Ospedaliero Universitaria Di Bologna, Bologna, Italy; 15 Department of Paediatrics, Infectious Diseases Unit, Regina Margherita Children’s Hospital, University of Turin, Turin, Italy; 16 Paediatric Clinic, University Hospital, Department of Medicine and Surgery, University of Parma, Parma, Italy; 17 Infectious Diseases Unit, Meyer Children’s Hospital IRCCS, Florence, Italy; 18 University Hospital Paediatric Department, Bambino Gesù Children’s Hospital, IRCCS, Rome, Italy; 19 Department of Pediatrics, Foundation IRCCS Policlinico San Matteo, University of Pavia, Pavia, Italy; 20 Pediatric Primary Care, National Pediatric Health Care System, Caserta, Italy; 21 Professor Emeritus of Pediatrics, University of Milan, Milan, Italy; 22 Department of Health Sciences, University of Florence, Viale Pieraccini 24, 50139 Firenze, Italy

**Keywords:** Pharyngitis, Streptococcus pyogenes, Sore throat, GAS, GABHS, Group A β-hemolytic streptococcus

## Abstract

This study aims to provide a comparison of the current recommendations about the management of acute pharyngitis. A literature search was conducted from January 2009 to 2023. Documents reporting recommendations on the management of acute pharyngitis were included, pertinent data were extracted, and a descriptive comparison of the different recommendations was performed. The quality of guidelines was assessed through the AGREE II instrument. Nineteen guidelines were included, and an overall moderate quality was found. Three groups can be distinguished: one group supports the antibiotic treatment of group A β-hemolytic Streptococcus (GABHS) to prevent acute rheumatic fever (ARF); the second considers acute pharyngitis a self-resolving disease, recommending antibiotics only in selected cases; the third group recognizes a different strategy according to the ARF risk in each patient. An antibiotic course of 10 days is recommended if the prevention of ARF is the primary goal; conversely, some guidelines suggest a course of 5–7 days, assuming the symptomatic cure is the goal of treatment. Penicillin V and amoxicillin are the first-line options. In the case of penicillin allergy, first-generation cephalosporins are a suitable choice. In the case of beta-lactam allergy, clindamycin or macrolides could be considered according to local resistance rates.

*    Conclusion*: Several divergencies in the management of acute pharyngitis were raised among guidelines (GLs) from different countries, both in the diagnostic and therapeutic approach, allowing the distinction of 3 different strategies. Since GABHS pharyngitis could affect the global burden of GABHS disease, it is advisable to define a shared strategy worldwide. It could be interesting to investigate the following issues further: cost-effectiveness analysis of diagnostic strategies in different healthcare systems; local genomic epidemiology of GABHS infection and its complications; the impact of antibiotic treatment of GABHS pharyngitis on its complications and invasive GABHS infections; the role of GABHS vaccines as a prophylactic measure. The related results could aid the development of future recommendations.

**Table:** 

**What is Known:** *• GABHS disease spectrum ranges from superficial to invasive infections and toxin-mediated diseases*. *• GABHS accounts for about 25% of sore throat in children and its management is a matter of debate*.	
**What is New:** *• Three strategies can be distinguished among current GLs: antibiotic therapy to prevent ARF, antibiotics only in complicated cases, and a tailored strategy according to the individual ARF risk*. *• The impact of antibiotic treatment of GABHS pharyngitis on its sequelae still is the main point of divergence; further studies are needed to achieve a global shared strategy*.

**Supplementary Information:**

The online version contains supplementary material available at 10.1007/s00431-023-05211-w.

## Introduction

Acute pharyngitis is a common event accounting for 2–5% of pediatric ambulatory visits, and it is one of the main reasons for prescribing antibiotics in children [1–3]. It is primarily due to viral infections and frequently sustained by adenovirus, Epstein-Barr virus, or Coxsackievirus [3].

Group A β-hemolytic Streptococcus (GABHS) accounts for about 25% of sore throat cases among children [4]. Its prevalence varies with the age: it is common in children older than 5, and the prevalence in adolescents varies from 19.3 to 30.1%. It has been considered rare in children younger than 3 years; however, data are contrasting: early studies investigating preschool children found that less than 10% of the ones younger than 3 years had a GABHS pharyngitis confirmed by an immune response [5, 6]; on the other hand, several studies reported a prevalence up to 28% of positive microbiological test in symptomatic children in the same age range [7, 8]. An etiological diagnosis solely based on a clinical investigation is challenging [9–11]. Some signs or symptoms can help discriminate between viral and bacterial pharyngitis (Table 1), but none of them is pathognomonic of GABHS pharyngitis.

**Table 1:** Clinical picture of infectious pharyngitis [3, 10]

	**Viral**	**Bacterial (*S. pyogenes*)**
Signs and symptoms	• Conjunctivitis • Coriza • Rhinorrhea • Cough • Diarrhea • Hoarseness • Oral ulcers/vesicles • Asthenia • Viral rash	• Fever • Tonsillar exudate • Palatal petechiae • Tender cervical nodes • Scarlet rash • Headache • Nausea • Vomit • Abdominal pain

Microbiological tests, such as culture, rapid antigen detection tests (RADT), and molecular tests based on polymerase chain reaction, are available as diagnostic tools. The culture test is the gold standard for the diagnosis of GABHS pharyngitis, but it has long turn-around times and considerable costs [12, 13]. None of the microbiological tests can distinguish a subject with GABHS pharyngitis from a carrier with intercurrent viral pharyngitis [9]. A GABHS carrier is defined by the identification of the pathogen in the pharynx without any symptoms or signs of infection [9]. Two meta-analyses reported a pooled prevalence of GABHS carriage of about 10% in asymptomatic children in high-income countries [4]. However, a retrospective cohort study assessed a carriage rate of up to 21% [14].

To better identify subjects with GABHS pharyngitis, clinical scoring systems have been proposed, including the McIsaac score and the FeverPAIN score (Table 2) [15, 16]. A meta-analysis compared the performance of Centor and McIsaac scores at diagnosing GABHS pharyngitis in children presenting to primary care. The two scores had equivalent performance characteristics; specifically, both were found to be sufficient to rule out GABHS infection in case of a score ≤ 0; conversely, neither score is sufficiently accurate to rule in it. Even with a score of 5, the positive predictive value was about 55% leading to the need for a point-of-care test to confirm the infection [17].

**Table 2:** Clinical scoring systems

**Centor score modified according to McIsaac **[15]		**FeverPAIN score **[16]	
**Clinic features**	**Score**	**Clinical features**	**Score**
Temperature > 38 °C	1	**Fever** in the last 24 h	1
Tender anterior cervical adenopathy	1	**P**urulence (Tonsillar exudates)	1
Tonsillar swelling or exudate	1	**A**ttend rapidly within 3 days due to the severity of symptoms	1
No cough	1	**I**nflamed tonsils (severe redness and swelling)	1
Age 5–14 years old	1	**N**o cough or coryza	1
Age 15–44 years old	0		
Age ≥ 45 years	− 1		

The management of GABHS pharyngitis is still a matter of debate, and the diagnostic and therapeutic approaches vary among guidelines. Different diagnostic strategies may result from economic evaluation depending on the healthcare system organization, as occurred for RADT in the UK [18, 19]. On the other hand, guidelines (GLs) are affected by local epidemiological factors; for instance, acute rheumatic fever (ARF) has been considered rare in Western countries since the end of the twentieth century, but it remains a substantial cause of morbidity and mortality in certain North American and Oceanian populations [20–22]. Moreover, a resurgence of ARF has been reported in the last 20 years in southern Europe, impacting acute pharyngitis management [20, 23].

These aspects can have an impact on diagnostic and therapeutic recommendations, resulting in different approaches even among high-income countries. The heterogeneity of GLs leads to confusion among healthcare professionals, inconsistency in the management of children with acute pharyngitis, and the low adherence to guidelines in clinical practice [24, 25]. A comprehensive perspective of the debate can be achieved by examining and comparing the different strategies. In 2011, our group summarized recommendations about the management of acute sore throat in national guidelines from Europe and North America [26]. It is recommended to update guidelines every 5 years to ensure their validity [27]. In the last decade, some national guidelines have been updated, and other new guidelines have been issued. Our aim was to provide an up-to-date discussion based on the latest guidance regarding the diagnosis and treatment of acute pharyngitis. Therefore, we carried out a thorough review of the literature, including the latest guidelines from Western countries.

## Materials and methods

A literature search was conducted from January 2009 to January 2023 through the following databases: MEDLINE, EMBASE, NICE: National Institute for Health and Care Excellence (www.nice.org.uk); Canadian CPG Infobase: Clinical Practice Guidelines Database (www.cma.ca/En/Pages/clinical-practice-guidelines.aspx); Scottish Intercollegiate Guidelines Network (SIGN) (www.sign.ac.uk); and Guidelines International Network (http://www.g-i-n.net/). Additional research was conducted on *Google*. The following search terms were used: pharyngitis, sore throat, tonsillitis, pharyngotonsillitis, *Streptococcus pyogenes*, group A b-hemolytic, and streptococcal pharyngitis*.*

Documents reporting recommendations on the diagnosis and treatment of acute pharyngitis were included. No language restriction was applied. References of all relevant articles were evaluated, and pertinent articles were included. Information about the diagnostic approach (use and interpretation of clinical score, rapid antigen test, and culture) and treatment (antibiotic regimen and duration of therapy) was extracted.

Two authors (ET and RP) independently assessed the quality of included guidelines through AGREE II instrument, which considers the following domains: “Scope and purpose,” “Stakeholder involvement,” “Rigour of development,” “Clarity of presentation,” “Applicability,” and “Editorial independence.” Each item of the domains was rated on a 7-point scale, and a domain score was calculated according to the AGREE II method [28]. We resolved any discrepancies through consensus.

## Results

Nineteen guidelines (GLs) were included, of these 10 are European (7 national [24, 29–35], 2 regional [36, 37], and one international [38]), 2 are national guidelines from the Oceania continent [39, 40], 6 are from North America (one from Canada [41] and 5 from the USA [9, 10, 42–44]), and one is from World Health Organization (WHO) [45].

The quality assessment results according to the AGREE II instrument are summarized in the Appendix. The GLs had a moderate quality overall. Scope and purpose were clearly stated in most GLs, with a median score of 97% (range 69–100%). Similarly, a high score was assigned for the clarity of presentation domain in most GLs with a median of 89% (range 58–100%). Systematic research of the literature was carried out in 10 GLs [29–31, 33, 36–38, 40, 42, 43], and 8 out of 19 clearly described the decision-making process used to state recommendations [29–32, 36–38, 42]; hence, the median score for the rigor of development domain was 54%. The lowest score was assigned for the applicability domain with a median score of 42% because facilitators and barriers to the application of GLs were rarely reported; the potential impact of the application of recommendations on resources was assessed only by 3 GLs [31, 36, 37], and audit instrument was mostly lacking [31, 36, 37, 42].

**Fig. 1:**
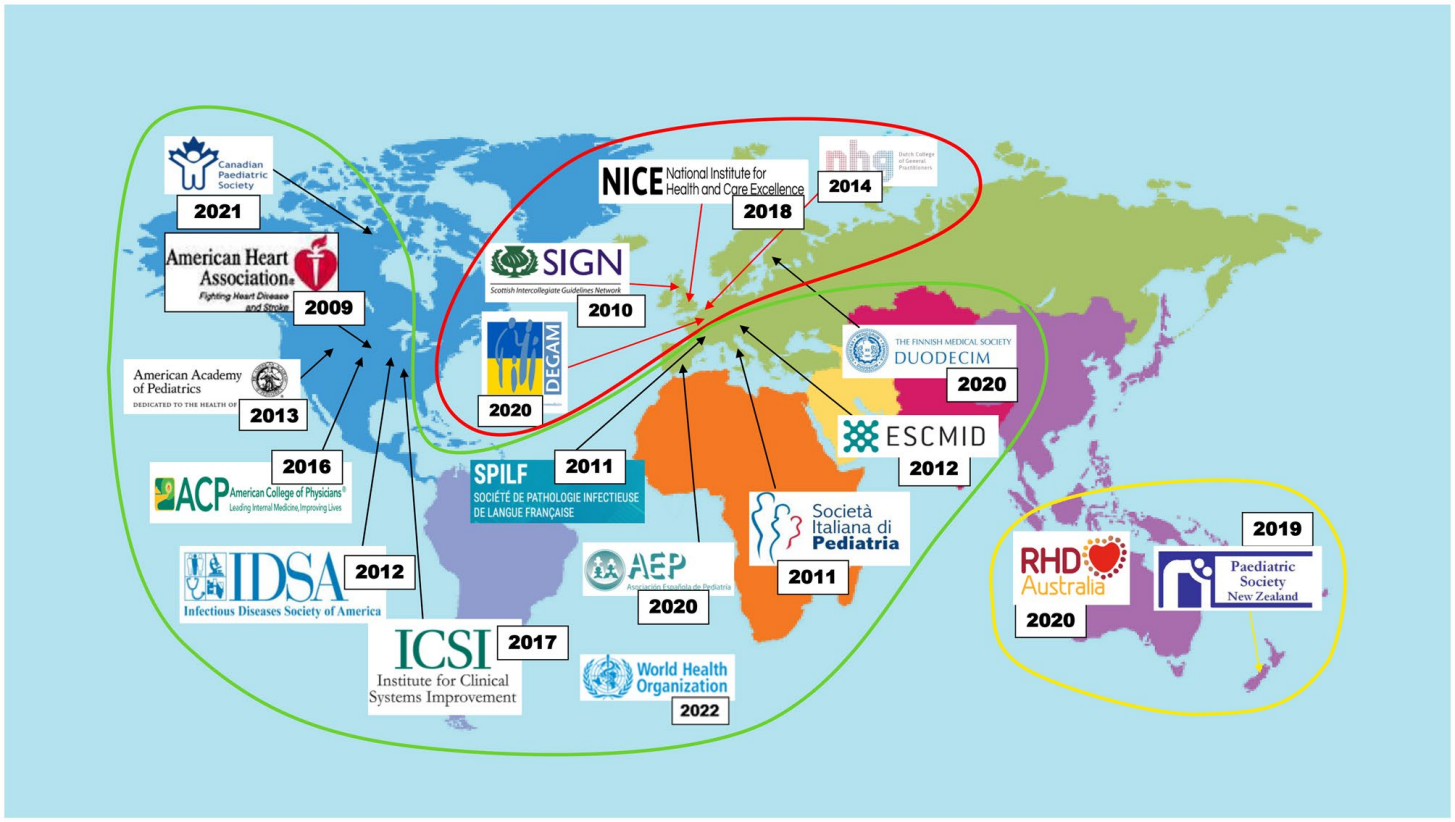
Geographical distribution of analyzed guidelines

The recommendations from each GL are summarized in Table 3. It is possible to distinguish current GLs into 3 groups (Fig. 1). One group, including GLs from WHO, North America, and most European countries, recommends the etiologic diagnosis of pharyngitis to correctly identify and treat GABHS pharyngitis in order to prevent acute rheumatic fever (ARF) and its cardiac complications [9, 10, 29, 32, 33, 35, 38, 41–45].

**Table 3:** Summary of analyzed guidelines

**Country years**	**Clinical score**	**Criteria for microbiological test**		**Criteria for antibiotic prescription**	**Antibiotic regimen**	**Treatment duration**	**Antibiotic regimen in case of penicillin allergy**
		**Rapid antigen detection test (RADT)**	**Cultural test**				
World Health Organization (WHO) 2022 [45]	Centor score	In countries with high incidence of ARF in case of Centor score 3–4	In countries with high incidence of ARF: - In case of Centor score 3–4 - In case of negative RADT and Centor score 3–4	In countries with medium or high risk of ARF: Centor score 3–4	Amoxicillin q 12 h Penicillin V q 6–8 h	High risk for ARF: 10 d Low risk for ARF: 5 d	Cephalexin or clarithromycin for 5 d
Germany 2021 [30]	McIsaac, or Centor, or FeverPAIN	If clinical score > 3 in children 3–15 years old	Not recommended	Clinical score > 4: immediate antibiotic therapy Clinical score 3: delayed prescription of antibiotic therapy * *Redeemed by the pt n case of worsening or persisting symptoms after 3–5 days	Penicillin V q 8 h	5–7 d	Clarithromycin for 5 d
UK NICE 2018 [31]	Fever PAIN or Centor score	Not routinely recommended		FeverPAIN 4–5 o Centor score 3–4: consider immediate antibiotic or backup prescription FeverPAIN 2–3: consider backup prescription	Penicillin V in 2 doses	5–10 d	Clarithromycin or erythromycin
Netherlands 2014 [24]	Not mentioned	Only in case of complication		Only in case of peritonsillar infiltrate, severe disease or high risk of complication	Penicillin V If peritonsillar infiltrate amoxicillin/clavulanate	Not mentioned	Not mentioned
Scotland SIGN 2010 [37]	Centor score	Not routinely recommended		Only in severe disease	Penicillin V q 6 h	10 d	Macrolides
Canada CPS 2021 [41]	Centor score	Centor score > 3	In region with high-incidence of ARF in case of negative RADT	Positive microbiological test (RADT or culture) In high-risk population, if testing is not available: Centor score > 3	Penicillin V in 2–3 doses or amoxicillin q 12–24 h	10 d	Clarithromycin or clindamycin for 10 d or azithromycin for 5 d
Spain 2020 [32]	FeverPAIN, McIsaac or Centor score	Regardless clinical score: - Children older than 3 y with suggestive symptoms, in absence of viral symptoms - Clinical suspicion of ARF or APSGN - Acute pharyngitis in pts with ARF or contacts of subjects with ARF - Household contact of a patient with recent APSGN - High-incidence of iGAS infections or recent contact with an affected pt - Household contact of a pt with acute pharyngitis and repeated intrafamilial transmission - Children younger than 3 y with suggestive symptoms of GABHS pharyngitis and close contact of a pt with confirmed GABHS pharyngitis	In case of negative RADT or if it is not available [63]	Positive microbiological test (RADT or culture)	Penicillina V q 12 h Amoxicillina q 8–12 h	10 d	Cefadroxil for 10 da Josamycin for 10 d
Finland 2020 [33, 34]	Centor score	Centor score ≥ 3	In case of symptoms persistence and negative RADT Recommended in case of an outbreak to evaluate antimicrobial sensitivity	Positive microbiological test (RADT or culture)	Penicillin V q 8 h	10 d	
US ICSI 2017 [42]	Centor score	Centor ≥ 3 and intention* to treat with antibiotics *The decision should be shared with the caregiver	If negative RADT in children	Positive microbiological test (RADT or culture)	Penicillin V or amoxicillin	10 days	Cephalexin or macrolides or clindamycin
US ACP/CDC 2016 [43]	Centor score	Centor ≥ 3		Positive microbiological test (RADT or culture)	Penicillin V q 6–12 h Amoxicillin q 12–24 h Penicillin G single dose	10 days	Cephalexin or cefadroxil Clindamycin or clarithromycin for 10 d or azithromycin for 5 d
Emilia Romagna (Italy) 2015 [36]	McIsaac	McIsaac 3–4	Not recommended	McIsaac 3–4 with positive RADT McIsaac 5 regardless RADT testing	Amoxicillin q 12 h	6 days	
US AAP 2013 [44]	McIsaac	McIsaac ≥ 2 Not to be performed in children under 3 years old		Positive microbiological test (RADT or culture)	Amoxicillin q 24 h	Not mentioned	Not mentioned
US IDSA 2012 [10]	Not mentioned	Children with suggestive symptoms Not to be performed under 3 years old or in case of viral symptoms	In children and adolescents with negative RADT	Positive microbiological test (RADT or culture)	Penicillin V q 6–12 h Amoxicillin q 12–24 h Penicillin G single dose	10 days	Cephalexin or cefadroxil Clindamycin or clarithromycin for 10 d or azithromycin for 5 d
Europe ESCMID 2012[38]	Centor McIsaac	Centor or McIsaac ≥ 3	Non routinely recommended	Centor score 3–4	Penicillin V q 8–12 h	10 days	Not mentioned
Italy 2011 [29]	McIsaac	McIsaac ≥ 2	Non routinely recommended	Positive microbiological test (RADT or culture)	Penicillin V or amoxicillin q 8–12 h Penicillin G single dose	10 days	Macrolides only if proven β-lactam antibiotics allergy
France 2011 [35]	McIsaac in adults	In children older than 3 years with suggestive symptoms In adults with McIsaac ≥ 2	Non routinely recommended	Positive microbiological test	Amoxicillin q 12 h	6 days	Cefotiam cefpodoxime or cefuroxime-axetil Macrolides
US AHA 2009 [9]	Not mentioned	Clinical suspicion of GAS pharyngitis		Positive microbiological test	Penicillin V q 8–12 h Amoxicillin q 24 h Penicillin G single dose	10 days	Cephalexin or cefadroxil Clindamycin or clarithromycin for 10 d or azithromycin for 5 d
Australia 2020 [39]	Not recommended in high-risk patients	Not recommended	In high-risk patients with suggestive symptoms perform a culture test only if follow-up is possible	High risk for ARF and pharyngitis: prescribe antibiotics Low-risk of ARF: only in case of positive microbiological test	Penicillin V q 12 h Penicillin G single dose	10 days	Cefalexin for 10 d Azithromycin for 5 d
New Zealand 2019 [40]	Not recommended in high-risk patients	Only in patients with low-risk of ARF	In high-risk patients with suggestive symptoms perform a culture test only if follow-up is possible	High-risk pts in case of clinical suspicion of GAS pharyngitis: prescribe empiric antibiotic therapy to stop in case of negative culture test Low-risk pts: prescribe antibiotics only in case of severe symptoms or occupational risk of spreading (e.g., healthcare teachers, students)	Penicillin V q 8–12 h Penicillin G single dose	10 days	Roxithromycin or erythromycin for 10 d

*WHO* World Health Organization, *ARF* acute rheumatic fever, *APSGN* acute post streptococcal glomerulonephritis, *iGAS* invasive group A streptococcal infections, *q* every, *d* days, *h* hours, *y* years, *pts* patients, *UK* United Kingdom, *NICE* National Institute of Care and Excellence, *SIGN* Scottish intercollegiate guidelines network, *US* United States of America, *ICSI* Institute for Clinical Systems Improvement, *ACP* America college of Physicians, *CDC* Centers for Disease Control and Prevention, *AAP* American Academy of Pediatrics, *IDSA* Infectious Diseases Society of America, *AHA* American Heart Association, *ESCMID* European Society of Clinical Microbiology and Infectious Diseases

The second group, including GLs from the UK, Germany, and the Netherlands, considers acute pharyngitis a benign and self-resolving disease, even if of streptococcal origin, and claims a limited effect of antibiotic therapy on disease length and suppurative complications rate. Consequently, they recommend antibiotics only in selected cases. Moreover, considering the low incidence of ARF in Western countries, the cost–benefit ratio of extensive use of antibiotic therapy is deemed unfavorable [24, 30, 31, 37].

A third group can be found considering Australian and New Zealand GLs, which, accounting for the high prevalence of ARF in their area, recognize two groups of patients according to the following risk factors: Maori or Aboriginal ethnicity, living in rural o remote areas, household overcrowding, low socioeconomic state and previous history of ARF in people aged 3–40 years old [39, 40].

### Diagnosis

#### Whom to test

According to the Spanish and French GLs and Infectious Disease Society of America (IDSA) recommendations, all children older than 3 years with clinical manifestation of pharyngitis should be tested for GABHS regardless of clinical score, aside from children with symptoms strongly suggestive of viral illness (Table 1) who should not be tested [10, 32, 35]. In the attempt to reduce false positive results (i.e., GABHS carriers with a viral sore throat) and inappropriate antibiotic treatment, WHO, Canadian, and most European and US GLs recommend using clinical scoring systems as a selection tool to identify patients to test. Specifically, WHO, Canadian, and Finnish GLs recommend using the Centor score, whereas the McIsaac score is recommended by Italian authors and the American Academy of Pediatrics (AAP) (Table 3) [29, 33, 36, 41, 44, 45]. In the absence of red flags (i.e., primary or secondary immunosuppression, sign of severe systemic disease or difficulty breathing, severe comorbidities, or increased risk of ARF), German GLs suggest discussing the option of starting an antibiotic therapy with the patient or the caregiver, and if it is considered, the treatment decision should be based on one of the 3 clinical scores [30].

On the other hand, in the National Institute for Health and Care Excellence (NICE) and the Scottish GLs, the diagnosis of GABHS pharyngitis relies exclusively on clinical scoring systems, and RADT is not recommended since they found that RADT does not improve antimicrobial prescribing or patient outcomes compared with clinical scoring tools alone [19, 31, 37].

#### How to test—microbiological test

Concerning RADT interpretation, most GLs consider a positive result sufficient for the diagnosis of GABHS pharyngitis due to its high specificity [10, 29, 32, 35, 38, 41]. Due to the variable sensitivity of RADT, in case of a negative result, US, Spanish, and WHO GLs recommend a confirmation culture test, particularly in children with a high clinical score [10, 32, 45]. Conversely, a routine confirmation culture test is not recommended according to Canadian and most European GLs, unless in case of persistent or worsening symptoms [29, 33, 38, 41]. Specifically, due to the very low prevalence of non-group-A streptococci pharyngitis in children, the German GL supports the use of RADTs, in children aged 3–15 years, only in the case of medium to high clinical probability of GABHS pharyngitis (≥ 3 score points). In the event of a negative result, no confirmation culture test is recommended [30]. NICE and the Scottish GLs do not recommend any microbiologic test [19, 31, 37].

According to Australiana and New Zealand GLs, a culture test should be obtained in children with risk factors for ARF and acute pharyngitis; in the latter case, empiric antibiotic treatment should be promptly started and stopped if the test is negative [39, 40]. RADT and clinical score systems are not recommended in high-risk patients; on the contrary, New Zealand GL suggests their use in low-risk populations to improve appropriate antibiotic prescription [39, 40].

All GLs agree that a microbiological test should not be recommended at the end of the treatment [9, 10, 29, 30, 32, 41]. Similarly, since the status of carrier bears a low risk of interindividual transmission and complications and it can persist from weeks to months [46], none of the GLs routinely recommend the screening for GABHS carriers or any antibiotic treatment [9, 10, 29, 32, 41]. Nonetheless, antibiotic treatment should be considered in GABHS carriers in populations at high risk of ARF and in specific circumstances such as community outbreaks of GABHS pharyngitis, ARF, acute post-streptococcal glomerulonephritis, or invasive GABHS infections [10, 33, 36, 39, 40, 42].

#### Laboratory tests

Blood tests (anti-streptolysin-O-titer, C-reactive protein, blood cell count) are not recommended by any GLs. Specific antibodies increase 3–8 weeks after infection and remain high for months, so they could be helpful for the diagnosis of non-suppurative complications but not for acute pharyngitis [9, 10, 29, 38, 41]. Increased levels of CRP or alterations of blood cell count are not specific and can be present even in the case of a viral infection.

### Treatment

#### When to treat—criteria for antibiotic prescription

Most European [26, 29, 32, 33, 35] and all North American [9, 42–44] GLs recommend antibiotic therapy only in case of a positive microbiological test. Canadian GL limits the previous recommendation to people with a low risk of ARF [41]. While in areas with medium to high risk of ARF, WHO and the Canadian Paediatric Society suggest prescribing antibiotics in patients with a Centor score of 3–4 without the need for a microbiological confirmation [41, 45] Likewise, the European Society of Clinical Microbiology and Infectious Diseases (ESCMID) recommends antibiotic therapy in all patients with a Centor score of 3–4 regardless ARF risk evaluation [38].

Clinical scoring systems are the basis of treatment prescription in NICE and German GLs in a shared decision-making process with the patient or caregiver. If antibiotic therapy is considered, a delayed prescription is recommended with a clinical score ≥ 3 and redeemed by the patient only in case of worsening or persisting symptoms after 3–5 days. However, an immediate antibiotic therapy should be provided in case of a high clinical score (Centor score 4, FeverPAIN, or McIsaac 4–5), although delayed prescription remains an option in this risk group [30, 31].

**Fig. 2:**
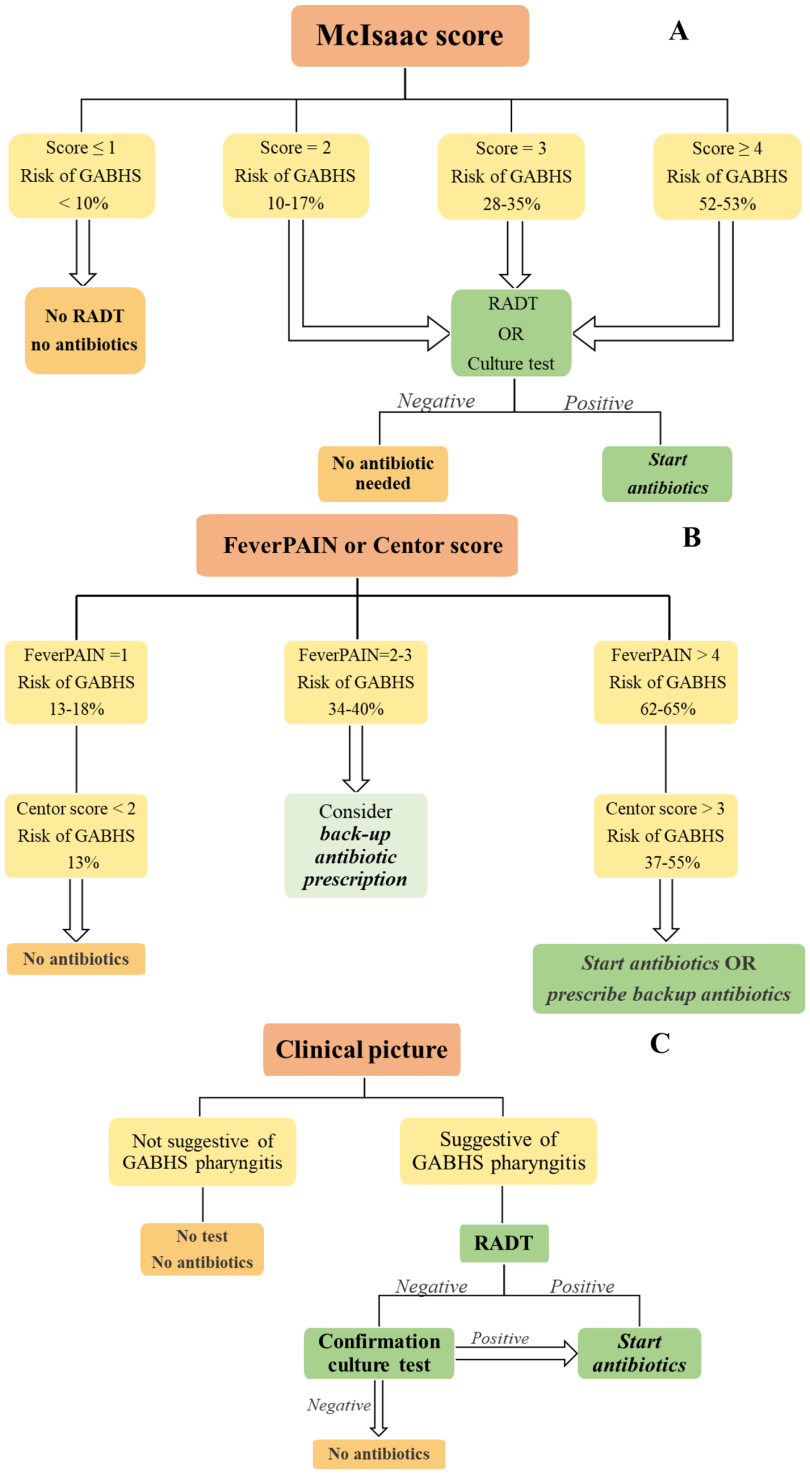
Management of pharyngitis according to Italian (**A**), NICE Guidelines 2018 (**B**), and IDSA Guidelines (**C**)

A mixed line is found in Italian regional GL from Emilia Romagna; considering the McIsaac score, they suggest treating all children with a score of 5 without the needing of a microbiological diagnosis and testing those with a score of 3–4, treating only those with a confirmed GABHS infection (Fig. 2) [36].

Based on local epidemiological data and the difficult follow-up in rural areas, Australian and New Zealand GLs recommend empirically treating all patients at high risk of ARF with a clinical suspicion of GABHS pharyngitis. Conversely, if a follow-up is possible, a culture test should be obtained before starting antibiotic therapy, and it should be stopped in case of a negative result [39, 40]. Whereas, low-risk patients should be treated only if a positive culture test is provided according to Australian GL [39].

Lastly, Dutch and Scottish GLs recommend prescribing antibiotics only in severe cases of pharyngitis or those complicated with peritonsillar infiltrate [24, 37].

#### How to treat—antibiotic regimen

All GLs agree in considering narrow-spectrum penicillin-based drugs as first-line options. If available, penicillin V is the drug of choice; otherwise, amoxicillin can be prescribed since it is equally effective and more palatable, making it a suitable option for children. When low adherence to treatment is suspected and follow-up is not possible, penicillin G, given in a single intramuscular dose, could be considered [9, 10, 29, 39, 40, 43]. Amoxicillin-clavulanate is recommended only by Dutch GLs in case of peritonsillar infiltrate [24].

In patients with a history of penicillin allergy, first-generation cephalosporins are suggested as an option [9, 10, 32, 39, 42, 43, 45]. In case of allergy to beta-lactam agents, macrolides could be considered [9, 10, 30–32, 35, 37, 39–43, 45]. However, the spread of macrolide-resistant GABHS isolates in Western countries must be taken into account; hence, it is advisable to consider local resistance rates and prove the susceptibility to macrolides if needed [29, 33]. Consequently, North American GLs consider clindamycin a possible alternative in case of penicillin allergy [9, 10, 41–43].

#### How long to treat—duration of therapy

The reduction of the probability of developing ARF in endemic settings is considered the only clear indication for antibiotic treatment by the WHO. Consequently, according to them, the local prevalence of ARF and individual risk factors should be assessed to establish the duration of therapy: in high-risk populations, GABHS pharyngitis should be treated for 10 days, while in low-risk ones, antibiotic treatment is indicated for 5 days or indeed withheld, even in cases of likely GABHS pharyngitis [45].

In line with that, most GLs still recommend a duration of antibiotic therapy of 10 days in an attempt to reduce ARF incidence [10, 29, 32, 33, 37–44]. On the other hand, given that their populations bear a low risk of ARF, German, English, and French GLs suggest a shorter course of antibiotics of 5–7 days, assuming the symptomatic cure is the primary goal of antibiotic treatment [30, 31, 35].

## Discussion

Acute pharyngitis is one of the main reasons for referring to a pediatric outpatient clinic, and it is one of the main reasons for prescribing antibiotics in children [1–3]. It is mainly of viral origin and only in one child out of four it is sustained by group A β-haemolytic Streptococcus (GABHS) infection [4]. The diagnosis and treatment of GABHS pharyngitis are still a matter of debate worldwide. Our review highlights the presence of some divergencies in the approach to acute sore throat among current GLs and the low quality in terms of rigor of development and applicability in most of them.

Comparing the included GLs, it is possible to detect two major areas of disagreement which are the diagnostic approach to GABHS pharyngitis and the role and regimen of the antibiotic therapy in this context.

Most GLs suggest a microbiological diagnosis of GABHS pharyngitis, recommending RADT in case of a high clinical score or suspicion [9, 10, 29, 30, 32, 33, 35, 36, 38, 41–43, 45]. It has been estimated that RADTs have a sensitivity of 82.9–94.6%, a specificity ranging from 84.9 to 99.1% [18] and a negative predictive value of 93.9% [12]. Hence, in case of a negative result, WHO, North American, and Spanish GLs recommend a confirmation culture test, and Finnish GLs recommend it in case of persisting symptoms [9, 10, 32, 33, 41–43, 45].

Conversely, English, Scottish, and Dutch GLs do not recommend using RADT at all, suggesting the antibiotic prescription only in case of a high clinical score (FeverPAIN > 4 or Centor > 3) or suppurative complications [19, 24, 31, 37]. This strategy is supported by a Health Technology Assessment conducted by the National Institute for Health Research (NIHR), showing that RADT was unlikely to be cost-effective in the English healthcare system compared to clinical scoring systems alone [18].

Point-of-care nucleic acid amplification tests (POC NAAT) are rising as a new diagnostic tool for GABHS pharyngitis in outpatient settings, but their role is not discussed in the current GLs. A recent cost-effectiveness analysis stated that POC NAATs have higher sensitivity than RADT and, in the USA, their use is less costly compared to a strategy based on RADT and culture confirmation [47] Further studies should be issued to define the actual role of this promising diagnostic tool.

Variations in local epidemiology, regional economy, and healthcare system organization affect the results of cost-effectiveness studies and their implications. Therefore, findings from such analysis could help the decision-making process throughout the GL development. Furthermore, health technology assessments could provide additional support for the selected diagnostic approach [16, 18].

Concerning therapy, antibiotic therapy for pharyngitis is controversial, and data about its efficacy in lowering complication rates are uncertain and primarily based on outdated studies. A Cochrane review found a reduction in suppurative complications in patients treated with antibiotics, but most of the analyzed studies were undertaken in the 1950s [48]. Similarly, RCTs published before 1975 showed a reduction in ARF incidence by up to two-thirds compared to placebo, in patients treated with intramuscular antibiotics during an outbreak. Since then, the incidence of ARF has decreased significantly in Western countries; therefore, this finding could not be confirmed in later studies due to the absence of ARF cases among both antibiotic-treated and control patients [48]. To date, data about national ARF incidence are lacking in high-income countries [49–51]. Therefore, trials investigating the incidence of suppurative and non-suppurative complications in high-income countries and how they could be influenced by antibiotic treatment should be issued to establish the actual role of antibiotic therapy as a primary prevention strategy.

Coates et al. estimated that enhancing primary and secondary prevention and tertiary services could avoid at least 74,000 deaths from rheumatic heart disease (RHD) in Africa in the next decade. Nevertheless, benefit–cost ratios and time-to-impact of primary prevention were low, though likely to increase over a long-time horizon through 2090 [52]. However, assumptions about the effects of primary prevention were affected by uncertain epidemiological data about GABHS pharyngitis in Africa, the estimation of a lower treatment coverage compared with other cost-effectiveness analyses, and the choice of a model which includes a formal healthcare evaluation for each sore throat that is not feasible in the analyzed setting [52, 53].

GABHS infections and their sequelae are one of the leading causes of antibiotic prescription. Data from US pediatricians showed that 60% of consultations for pharyngitis result in antibiotics prescriptions, even if it is primarily of viral origin [54]. This is in line with European and US data; notably, a survey among Italian primary-care pediatricians revealed that only 8% adhere to national GLs [25, 55–58]. Moreover, the prescribed antibiotic regimen is not consistent with GLs. An Italian study assessing pharyngitis management in outpatient settings showed that cephalosporins were largely prescribed in non-GABHS pharyngitis and if no microbiological result was available children were equally likely to receive broad or narrow-spectrum antibiotics [1].

The overuse of antibiotics for acute pharyngitis, particularly broad-spectrum ones, is even more concerning given the recent identification of GABHS strains with reduced susceptibility to β-lactam [59]. In 2017–2018, within a community outbreak in Seattle, two emm43.4 GABHS isolates with eightfold reduced susceptibility to both amoxicillin and ampicillin were identified, and a missense mutation in penicillin-binding proteins (PBPs) was detected and identified as PBP2 × . [60] Since then, further PBP2 × mutant isolates have been reported; however, no change in in vivo virulence seems to be demonstrated [59]. Nonetheless, these findings are consistent with first steps in β-lactam resistance development. On the other hand, GABHS strains resistant to macrolides and clindamycin are increasing, resulting in infection recurrence, treatment failure, and poor outcomes. It is a consequence of ribosomal target site modification in GABHS and is associated with several *emm-*types. Thus, it is of primary importance to monitor isolates susceptibility and take it into account in future treatment recommendations. Moreover, genomic epidemiological studies could support the choice of the most appropriate second-line antibiotic regimen in different countries [59].

GABHS disease spectrum is broad, spanning from superficial infection (pharyngitis, impetigo) to invasive infections (sepsis, abscess, cellulitis) and toxin-mediated diseases (necrotizing fasciitis, streptococcal toxic shock syndrome); however, whether these diseases share the same transmission network is yet to be fully understood. Through whole genome sequencing, Li et al. characterized GABHS strains isolated from patients with pharyngitis and invasive infections from a restricted region, finding 97 genomically closely related isolates. Of these genomic clusters, 30 contained isolates from pharyngitis and invasive and toxin-mediated diseases, suggesting a common transmission route [61]. Pharyngitis in children might be the most likely initial source of invasive genotypes, so a proper treatment could help reduce the circulation of invasive clusters.

To date, antibiotics are the only treatment and prophylactic intervention against GABHS infection and its sequelae. Despite many years of research, an authorized vaccine against GABHS is not available yet due to the extensive genetic diversity of the pathogen, potential autoimmune epitopes, and the fact that it is an exclusively human-adapted pathogen, making it challenging to use animal models [59, 62]. However, GABHS vaccine research and development have been declared a priority of the WHO 2018 global resolution on ARF and RHD, and some vaccines targeting the M-protein have reached phase I in clinical trials [59]. Hence, advances in this field could change future recommendations in the management of sore throat and help reduce antibiotic prescriptions and antimicrobial resistance.

## Limitations

Some documents reporting recommendations about the diagnosis and treatment of acute pharyngitis might have been missed.

## Conclusion

This review highlights several divergencies in the diagnostic and treatment approach to acute pharyngitis in GLs from different countries. In our opinion, it is advisable to define a common strategy based on local epidemiological data since the management of GABHS pharyngitis could affect the global burden of GABHS disease. The following issues should be addressed in future research and considered to develop forthcoming recommendations:

- The optimal duration of antibiotic treatment of GABHS pharyngitis and its impact on suppurative and non-suppurative complications and on the rate of recurrent and invasive streptococcal infections
- Cost-effectiveness analysis of available diagnostic tools and strategies in different healthcare systems in order to reduce inappropriate antibiotic therapies
- Local epidemiology of GABHS infection and its complications, including genomic epidemiology reporting *emm*-types and antibiotic-resistance rates
- Advances in GABHS vaccine development and its role in GABHS-related disease prevention

### Supplementary Information

Below is the link to the electronic supplementary material.

Supplementary file1 (DOCX 18 KB)

## Abbreviations

AAP: American Academy of Pediatrics
ACP: American College of Physicians
AHA: American Heart Association
ARF: Acute rheumatic fever
CDC: Centers for Disease Control and Prevention
CRP: C-reactive protein
d: Days
ESCMID: European Society of Clinical Microbiology and Infectious Diseases
GABHS: Group A β-hemolytic Streptococcus
GL: Guideline
h: Hours
ICSI: Institute for Clinical Systems Improvement
IDSA: Infectious Diseases Society of America
NICE: National Institute for Health and Care Excellence
PBPs: Penicillin-binding proteins
POC NAAT: Point-of-care nucleic acid amplification tests
q: Every
RADT: Rapid antigen detection test
RHD: Rheumatic heart disease
SIGN: Scottish intercollegiate guidelines network
UK: United Kingdom
US: United States of America
WHO: World Health Organization

## References

[1] Barbieri E, Donà D, Cantarutti A, Lundin R, Scamarcia A, Corrao G (2019). Antibiotic prescriptions in acute otitis media and pharyngitis in Italian pediatric outpatients. Ital J Pediatr.

[2] Dona D, Baraldi M, Brigadoi G, Lundin R, Perilongo G, Hamdy RF (2018). The impact of clinical pathways on antibiotic prescribing for acute otitis media and pharyngitis in the emergency department. Pediatr Infect Dis J.

[3] Sykes EA, Wu V, Beyea MM (2020). Pharyngitis: approach to diagnosis and treatment. Can Fam Physician.

[4] Oliver J, Malliya Wadu E, Pierse N, Moreland NJ, Williamson DA, Baker MG (2018) Group A Streptococcus pharyngitis and pharyngeal carriage: a meta-analysis. PLoS Negl Trop Dis 12:e0006335. 10.1371/journal.pntd.0006335 10.1371/journal.pntd.0006335 PMC5875889 29554121

[5] Nussinovitch M, Finkelstein Y, Amir J, Varsano I (1999). Group A beta-hemolytic streptococcal pharyngitis in preschool children aged 3 months to 5 years. Clin Pediatr (Phila).

[6] Amir J, Shechter Y, Eilam N, Varsano I (1994). Group A beta-hemolytic streptococcal pharyngitis in children younger than 5 years. Isr J Med Sci.

[7] Mendes N, Miguéis C, Lindo J, Gonçalves T, Miguéis A (2021). Retrospective study of group A Streptococcus oropharyngeal infection diagnosis using a rapid antigenic detection test in a paediatric population from the central region of Portugal. Eur J Clin Microbiol Infect Dis Off Publ Eur Soc Clin Microbiol.

[8] Shaikh N, Leonard E, Martin JM (2010). Prevalence of streptococcal pharyngitis and streptococcal carriage in children: a meta-analysis. Pediatrics.

[9] Gerber MA, Baltimore RS, Eaton CB, Gewitz M, Rowley AH, Shulman ST (2009). Prevention of rheumatic fever and diagnosis and treatment of acute streptococcal pharyngitis. Circulation.

[10] Shulman ST, Bisno AL, Clegg HW, Gerber MA, Kaplan EL, Lee G (2012). Clinical practice guideline for the diagnosis and management of group A Streptococcal pharyngitis: 2012 update by the Infectious Diseases Society of America. Clin Infect Dis.

[11] Kimberlin D, Barnett ED, Lynfield R, Sawyer MH. American Academy of Pediatrics. Group A streptococcal infections. Red Book. Red Book (2021). Report of the Commitee on Infectious Disease. Itasca.

[12] Cohen JF, Pauchard J-Y, Hjelm N, Cohen R, Chalumeau M (2020) Efficacy and safety of rapid tests to guide antibiotic prescriptions for sore throat. Cochrane Database Syst Rev 6:CD012431. 10.1002/14651858.CD012431.pub2 10.1002/14651858.CD012431.pub2 PMC7271976 32497279

[13] Mustafa Z, Ghaffari M (2020) Diagnostic methods, clinical guidelines, and antibiotic treatment for group A streptococcal pharyngitis: a narrative review. Front Cell Infect Microbiol 10:563627. 10.3389/fcimb.2020.563627 10.3389/fcimb.2020.563627 PMC7593338 33178623

[14] Rick A-M, Zaheer HA, Martin JM (2020). Clinical features of group A Streptococcus in children with pharyngitis: carriers versus acute infection. Pediatr Infect Dis J.

[15] McIsaac WJ, White D, Tannenbaum D, Low DE (1998). A clinical score to reduce unnecessary antibiotic use in patients with sore throat. CMAJ Can Med Assoc J.

[16] Little P, Moore M, Hobbs FDR, Mant D, McNulty C, Williamson I et al (2013) Primary care Streptococcal Management (PRISM) study: identifying clinical variables associated with Lancefield group A β-haemolytic streptococci and Lancefield non-group A streptococcal throat infections from two cohorts of patients presenting with an acute sore throat. BMJ Open 3:e003943. 10.1136/bmjopen-2013-003943 10.1136/bmjopen-2013-003943 PMC3808825 24163209

[17] Willis BH, Coomar D, Baragilly M (2020). Comparison of Centor and McIsaac scores in primary care: a meta-analysis over multiple thresholds. Br J Gen Pract J R Coll Gen Pract.

[18] Fraser H, Gallacher D, Achana F, Court R, Taylor-Phillips S, Nduka C (2020). Rapid antigen detection and molecular tests for group A streptococcal infections for acute sore throat: systematic reviews and economic evaluation. Health Technol Assess.

[19] National Institute for Health and Care Excellence (2019) Rapid tests for group A streptococcal infections in people with a sore throat (DG38). www.nice.org.uk/guidance/dg38

[20] Liang Y, Yu D, Lu Q, Zheng Y, Yang Y (2023) The rise and fall of acute rheumatic fever and rheumatic heart disease: a mini review. Front Cardiovasc Med 10 10.3389/fcvm.2023.1183606 PMC10242100 37288267

[21] Gordon J, Kirlew M, Schreiber Y, Saginur R, Bocking N, Blakelock B, Haavaldsrud M, Kennedy C, Farrell T (2015) Acute rheumatic fever in First Nations communities in northwestern Ontario: Social determinants of health "bite the heart". Can Fam Physician 61:881–886 PMC4607335 26759842

[22] Bradley-Hewitt T, Longenecker CT, Nkomo V, Osborne W, Sable C, Scheel A (2019). Trends and presentation patterns of acute rheumatic fever hospitalisations in the United States. Cardiol Young.

[23] Fabi M, Calicchia M, Miniaci A, Balducci A, Tronconi E, Bonetti S (2019). Carditis in acute rheumatic fever in a high-income and moderate-risk country. J Pediatr.

[24] de Jongh E, Opstelten W (2015) Werkgroep NHG-Standaard Acute keelpijn. [Revision of the Dutch College of General Practitioners practice guideline ’Acute sore throat’]. Ned Tijdschr Geneeskd 159:A9456 26332822

[25] Hek K, van Esch TEM, Lambooij A, Weesie YM, van Dijk L (2020). Guideline adherence in antibiotic prescribing to patients with respiratory diseases in primary care: prevalence and practice variation. Antibiotics.

[26] Chiappini E, Regoli M, Bonsignori F, Sollai S, Parretti A, Galli L (2011). Analysis of different recommendations from international guidelines for the management of acute pharyngitis in adults and children. Clin Ther.

[27] Vernooij RW, Sanabria AJ, Solà I, Alonso-Coello P, Martínez GL (2014). Guidance for updating clinical practice guidelines: a systematic review of methodological handbooks. Implement Sci IS.

[28] Brouwers M, Kho M, Browman G, Burgers J, Cluzeau F, Feder G et al (2010) AGREE II: advancing guideline development, reporting and evaluation in healthcare. 182:E839–842 10.1503/cmaj.090449 PMC3001530 20603348

[29] Chiappini E, Principi N, Mansi N, Serra A, De Masi S, Camaioni A (2012). Management of acute pharyngitis in children: summary of the Italian National Institute of Health guidelines. Clin Ther.

[30] Krüger K, Töpfner N, Berner R, Windfuhr J, Oltrogge JH (2021) Guideline group. Clinical practice guideline: sore throat. Dtsch Arzteblatt Int 118:arztebl.m2021.0121. 10.3238/arztebl.m2021.0121 10.3238/arztebl.m2021.0121 PMC8245861 33602392

[31] National Institute for Health and Care Excellence (2018) Sore throat (acute): antimicrobial prescribing [NG84]. https://www.nice.org.uk/guidance/ng84 . Accessed 26 Apr 2022

[32] Piñeiro Pérez R, Álvez González F, Baquero-Artigao F, Cruz Cañete M, de la Flor i Bru J, Fernández Landaluce A et al (2020) Actualización del documento de consenso sobre el diagnóstico y tratamiento de la faringoamigdalitis aguda. An Pediatría 93:206.e1–206.e8. 10.1016/j.anpedi.2020.05.004 10.1016/j.anpedi.2020.05.004 32605870

[33] Finnish Medical Society Duodecim, Finnish Association for Central Practice, Finnish Otolaryngological Society, Infectious Diseases Society of Finland, Clinical Microbiologists Society (2020) Sore throat. Current Care Summary. https://www.kaypahoito.fi/en/ccs00095 . Accessed 29 Apr 2022

[34] Duodecim. Faringite (2020) Raccomandazione di cura attuale. https://www.kaypahoito.fi/hoi38020#s12 . Accessed 29 Apr 2022

[35] Cohen R, Azria R, Barry B, Bingen E, Cavallo J-D, Chidiac C et al (2011) Antibiotherapie par voie generale en pratique courante dans les infections respiratoires hautes de l’adulte et l’enfant. https://www.infectiologie.com/UserFiles/File/medias/Recos/2011-infections-respir-hautes-recommandations.pdf . Accessed 29 Apr 2022

[36] Agenzia sanitaria e sociale regionale dell’Emilia-Romagna (2015) Faringotonsillite in età pediatrica Linea guida regionale. http://assr.regione.emilia-romagna.it/it/servizi/pubblicazioni/dossier/doss253 . Accessed 7 Apr 2022

[37] Scottish Intercollegiate Guidelines Network (2010) Management of sore throat and indications for tonsillectomy: a national clinical guideline. https://www.sign.ac.uk/media/1055/sign117.pdf . Accessed 29 Apr 2022

[38] Pelucchi C, Grigoryan L, Galeone C, Esposito S, Huovinen P, Little P (2012). Guideline for the management of acute sore throat. Clin Microbiol Infect.

[39] RHDAustralia. The 2020 Australian guideline for prevention, diagnosis and management of acute rheumatic fever and rheumatic heart disease (3rd edition) 2020. https://www.rhdaustralia.org.au/arf-rhd-guideline 10.5694/mja2.50851 33190309

[40] National Heart Foundation of New Zealand (2019) Group A streptococcal sore throat management guideline. 2019 Update. Auckland. https://www.heartfoundation.org.nz/resources/group-a-streptococcal-sore-throat-management . Accessed 5 May 2022

[41] Canadian Paediatric Society (2021) Group A streptococcal (GAS) pharyngitis: a practical guide to diagnosis and treatment | Canadian Paediatric Society. https://cps.ca/en/documents//position//group-a-streptococcal/ . Accessed 29 Apr 2022

[42] Short S, Bashir H, Marshall P, Miller N, Olmschenk D, Prigge K et al (2017) Diagnosis and treatment of respiratory illness in children and adults. https://www.icsi.org/wp-content/uploads/2019/01/RespIllness.pdf . Accessed 5 May 2022

[43] Harris AM, Hicks LA, Qaseem A (2016). Appropriate antibiotic use for acute respiratory tract infection in adults: advice for high-value care from the American College of Physicians and the Centers for Disease Control and Prevention. Ann Intern Med.

[44] Hersh AL, Jackson MA, Hicks LA, Committee on Infectious Diseases, Brady MT, Byington CL et al (2013) Principles of judicious antibiotic prescribing for upper respiratory tract infections in pediatrics. Pediatrics 132:1146–54. 10.1542/peds.2013-3260 10.1542/peds.2013-3260 24249823

[45] World Health Organization (2022) The WHO AWaRe (Access, Watch, Reserve) antibiotic book

[46] Wessels MR (2011). Streptococcal Pharyngitis. N Engl J Med.

[47] Bilir SP, Kruger E, Faller M, Munakata J, Karichu JK, Sickler J et al (2021) US cost-effectiveness and budget impact of point-of-care NAAT for streptococcus. Am J Manag Care 27:e157–63. 10.37765/ajmc.2021.88638 10.37765/ajmc.2021.88638 34002967

[48] Spinks A, Glasziou PP, Del Mar CB (2021) Antibiotics for treatment of sore throat in children and adults. Cochrane Database Syst Rev 12:CD000023. 10.1002/14651858.CD000023.pub5 10.1002/14651858.CD000023.pub5 PMC8655103 34881426

[49] Marino A, Cimaz R, Pelagatti MA, Tattesi G, Biondi A, Menni L et al (2021) Acute rheumatic fever: where do we stand? An epidemiological study in Northern Italy. Front Med 8:621668. 10.3389/fmed.2021.621668 10.3389/fmed.2021.621668 PMC7943448 33718402

[50] Alberio AMQ, Pieroni F, Di Gangi A, Cappelli S, Bini G, Abu-Rumeileh S et al (2021) Toward the knowledge of the epidemiological impact of acute rheumatic fever in Italy. Front Pediatr 9:746505. 10.3389/fped.2021.746505 10.3389/fped.2021.746505 PMC8714836 34976887

[51] Breda L, Marzetti V, Gaspari S, Torto MD, Chiarelli F, Altobelli E (2012). Population-based study of incidence and clinical characteristics of rheumatic fever in Abruzzo, Central Italy, 2000–2009. J Pediatr.

[52] Coates MM, Sliwa K, Watkins DA, Zühlke L, Perel P, Berteletti F (2021). An investment case for the prevention and management of rheumatic heart disease in the African Union 2021–30: a modelling study. Lancet Glob Health.

[53] Okello E, Beaton A (2021). Targeted investment needed to end rheumatic heart disease in Africa. Lancet Glob Health.

[54] Dooling KL, Shapiro DJ, Van Beneden C, Hersh AL, Hicks LA (2014). Overprescribing and inappropriate antibiotic selection for children with pharyngitis in the United States, 1997–2010. JAMA Pediatr.

[55] Di Muzio I, d’Angelo DM, Di Battista C, Lapergola G, Zenobi I, Marzetti V (2020). Pediatrician’s approach to diagnosis and management of group A streptococcal pharyngitis. Eur J Clin Microbiol Infect Dis.

[56] de Bie S, Kaguelidou F, Verhamme KMC, De Ridder M, Picelli G, Straus SMJM (2016). Using prescription patterns in primary care to derive new quality indicators for childhood community antibiotic prescribing. Pediatr Infect Dis J.

[57] Norton LE, Lee BR, Harte L, Mann K, Newland JG, Grimes RA et al (2018) Improving guideline-based streptococcal pharyngitis testing: a quality improvement initiative. Pediatrics 142:e20172033. 10.1542/peds.2017-2033 10.1542/peds.2017-2033 29925574

[58] Smith DRM, Dolk FCK, Pouwels KB, Christie M, Robotham JV, Smieszek T (2018) Defining the appropriateness and inappropriateness of antibiotic prescribing in primary care. J Antimicrob Chemother 73:ii11–8. 10.1093/jac/dkx503 10.1093/jac/dkx503 PMC5890733 29490061

[59] Brouwer S, Rivera-Hernandez T, Curren BF, Harbison-Price N, De Oliveira DMP, Jespersen MG et al (2023) Pathogenesis, epidemiology and control of group A Streptococcus infection. Nat Rev Microbiol 1–17. 10.1038/s41579-023-00865-7 10.1038/s41579-023-00865-7 PMC9998027 36894668

[60] Vannice KS, Ricaldi J, Nanduri S, Fang FC, Lynch JB, Bryson-Cahn C (2020). Streptococcus pyogenes pbp2x mutation confers reduced susceptibility to β-lactam antibiotics. Clin Infect Dis.

[61] Li Y, Dominguez S, Nanduri SA, Rivers J, Mathis S, Li Z (2022). Genomic characterization of group A Streptococci causing pharyngitis and invasive disease in Colorado, USA, June 2016- April 2017. J Infect Dis.

[62] Vekemans J, Gouvea-Reis F, Kim JH, Excler J-L, Smeesters PR, O’Brien KL (2019). The path to group a Streptococcus vaccines: World Health Organization research and development technology roadmap and preferred product characteristics. Clin Infect Dis.

[63] Piñeiro Pérez R, Hijano Bandera F, Alvez González F, Fernández Landaluce A, Silva Rico JC, Pérez Cánovas C (2003) Consensus document on the diagnosis and treatment of acute tonsillopharyngitis An Pediatr Barc Spain 7(5):342.e1 13 10.1016/j.anpedi.2011.07.015 10.1016/j.anpedi.2011.07.015 PMC7105079 21920830

