# Supplementary material for: Acute pharyngitis in children and adults: descriptive comparison of current recommendations from national and international guidelines and future perspectives
Source: Eur J Pediatr. 2023 Oct 11;182(12):5259–73. doi: 10.1007/s00431-023-05211-w (PMC10746578; doi:10.1007/s00431-023-05211-w)
Supplement: Supplementary file 1 — Supplementary file1 (DOCX 18 KB) [file 431_2023_5211_MOESM1_ESM.docx]

**APPENDIX**

Table-A1: Domain-standardised scores of guidelines assessed through the AGREE II instrument.

|  | Scope and purpose | Stakeholder involvement | Rigour of development | Clarity of presentation | Applicability | Editorial Independence |
| --- | --- | --- | --- | --- | --- | --- |
| Germany 2021 | 89% | 69% | 84% | 89% | 48% | 100% |
| NICE 2018 | 100% | 78% | 96% | 100% | 100% | 100% |
| Netherlands 2014 | 86% | 39% | 6% | 86% | 13% | 54% |
| SIGN 2010 | 100% | 100% | 94% | 100% | 90% | 100% |
| Canada 2021 | 69% | 44% | 13% | 72% | 21% | 0% |
| Finland 2020 | 94% | 97% | 34% | 58% | 17% | 83% |
| US ICSI 2017 | 100% | 92% | 94% | 100% | 56% | 100% |
| US ACP/CDC 2016 | 86% | 50% | 47% | 89% | 35% | 100% |
| US AAP 2013 | 92% | 58% | 39% | 58% | 8% | 88% |
| US IDSA 2012 | 64% | 39% | 6% | 72% | 0% | 42% |
| US AHA 2009 | 89% | 56% | 42% | 83% | 33% | 100% |
| ESCMID 2012 | 100% | 39% | 73% | 89% | 58% | 100% |
| Italy 2011 | 100% | 56% | 65% | 100% | 25% | 100% |
| France 2011 | 100% | 56% | 35% | 78% | 58% | 0% |
| Spain 2020 | 89% | 50% | 44% | 100% | 33% | 67% |
| Australia 2020 | 100% | 78% | 60% | 100% | 71% | 50% |
| New Zealand 2019 | 100% | 78% | 88% | 100% | 75% | 100% |
| Emilia Romagna 2015 | 100% | 100% | 100% | 100% | 100% | 100% |
| *Median score* | ***97%*** | ***57%*** | ***54%*** | ***89%*** | ***42%*** | ***100%*** |

**Abbreviations**: NICE: National Institute of Care and Excellence; SIGN: Scottish intercollegiate guidelines network; US: United States of America; ICSI: Institute for Clinical Systems Improvement; ACP: America college of Physicians; CDC: Centers for Disease Control and Prevention; AAP: American Academy of Pediatrics; IDSA: Infectious Diseases Society of America; AHA: American Heart Association; ESCMID: European Society of Clinical Microbiology and Infectious Diseases.
